# The role of microRNAs in the molecular link between circadian rhythm and autism spectrum disorder

**DOI:** 10.1080/19768354.2023.2180535

**Published:** 2023-02-23

**Authors:** Ji Young Kim, Wanil Kim, Kyung-Ha Lee

**Affiliations:** a Department of Molecular Biology, Pusan National University, Busan, Republic of Korea; b Department of Biochemistry, College of Medicine, Gyeongsang National University, Jinju-si, Republic of Korea

**Keywords:** Circadian rhythm, MicroRNA, autism spectrum disorder

## Abstract

Circadian rhythm regulates physiological cycles of awareness and sleepiness. Melatonin production is primarily regulated by circadian regulation of gene expression and is involved in sleep homeostasis. If the circadian rhythm is abnormal, sleep disorders, such as insomnia and several other diseases, can occur. The term ‘autism spectrum disorder (ASD)’ is used to characterize people who exhibit a certain set of repetitive behaviors, severely constrained interests, social deficits, and/or sensory behaviors that start very early in life. Because many patients with ASD suffer from sleep disorders, sleep disorders and melatonin dysregulation are attracting attention for their potential roles in ASD. ASD is caused by abnormalities during the neurodevelopmental processes owing to various genetic or environmental factors. Recently, the role of microRNAs (miRNAs) in circadian rhythm and ASD have gained attraction. We hypothesized that the relationship between circadian rhythm and ASD could be explained by miRNAs that can regulate or be regulated by either or both. In this study, we introduced a possible molecular link between circadian rhythm and ASD. We performed a thorough literature review to understand their complexity.

## Relationship between circadian rhythms and ASD

### Circadian rhythm

The day-night cycle affects almost all living creatures on Earth, and their physiological functions follow a roughly 24-hour cyclic pattern. The term ‘circadian rhythm’ refers to this physiological, 24-hour cycle that exhibits an endogenous and entrainable oscillation (Dibner et al. 2010; Patke et al. 2020). *In vivo*, circadian rhythms are mainly regulated by environmental signals such as light, food, and arousal stimuli (Lee and Kim 2021). Circadian rhythm is generated by the molecular clock system. Notably, the mammalian circadian clock is conceptualized as a hierarchical system in which the brain clock located in the suprachiasmatic nucleus acts as a master regulator that synchronizes or tunes other peripheral clocks distributed throughout the body (Takahashi 2017). Circadian rhythm plays an important role in the central nervous system and has a great impact on the physiology of organisms. It is important to maintain the appropriate circadian rhythm to maintain homeostasis; dysregulated circadian rhythms can increase the potential of developing dangerous diseases such as cancer and can have a notable impact on the development of brain diseases such as degenerative neurological diseases (Sulli et al. 2019; Shin 2020). Variations in light are captured by the optic nerves that feed the signals in the brain, thereby, forming the circadian rhythm that is essential for day-to-day life (Sulli et al. 2018).

Sleep homeostasis is linked to the 24-hour cycle and is controlled by melatonin. Melatonin is a powerful antioxidant molecule involved in the regulation of the 24-hour cycle, seasonal rhythms, and immune functions (Brzezinski 1997). Patients with Magenis syndrome often show symptoms of autism spectrum disorder (ASD). And patients with Megenis syndrome have sleep disturbance as one of their major problems (Trickett et al. 2018). And in patients with ASD, abnormal concentrations of melatonin have been observed and are thought to impact human behavior (Trickett et al. 2018; Wu et al. 2020b; Martinez-Cayuelas et al. 2022).

### Autism spectrum disorder

Autism spectrum disorder is a prevalent, highly heritable, and heterogeneous neurodevelopmental disorder with underlying cognitive characteristics and frequently co-occurs with other illnesses. Manifestations of autism include difficulties with social interaction and communication, abnormal sensory experiences, repetitive behaviors, and varied degrees of intellectual disability. Along with these core symptoms, co-occurring psychiatric or neurological disorders are frequent in individuals with autism, with attention-deficit/hyperactivity disorder (ADHD), anxiety, depression, and epilepsy being the most common (Kas et al. 2014; Herrero et al. 2020; Lord et al. 2020; Park and Jung 2022).

Over the last decade, considerable research has been conducted to identify the causes of ASD, and great progress has been made in understanding the genetics of ASD. The ASD probability that may occur as a genetic factor is estimated to be 17% to 52% of the total incidence of ASD (Iakoucheva et al. 2019; Zhou et al. 2019). New mutations have been identified, including copy number modifications and point mutations that are likely to disrupt protein-coding genes and lead to ASD (Rylaarsdam and Guemez-Gamboa 2019; Dell'Osso et al. 2022). Several studies have focused on the role of the 24-hour periodic rhythm in ASD since aberrant melatonin levels, patterns, and sleep problems have been associated with the ASD (Melke et al. 2008; Doyen et al. 2011; Rossignol and Frye 2011; Wu et al. 2020b; Martinez-Cayuelas et al. 2022).

### Relationship between circadian rhythm and ASD

**Figure 1.**
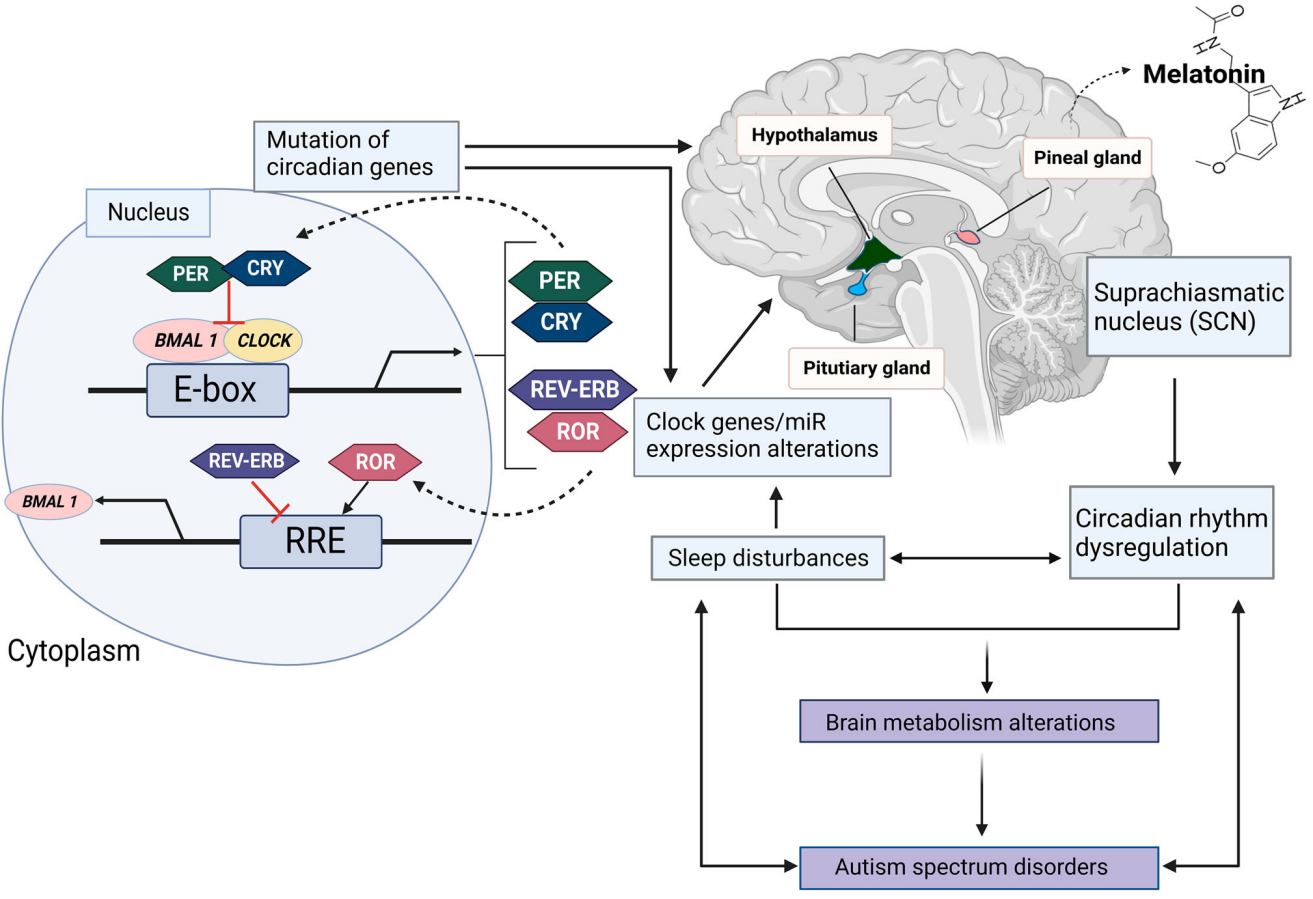
The mechanism of circadian rhythm and ASD. Explaining the molecular mechanism of the circadian clock system and the relationship between circadian rhythms and ASD. The core clock genes, BMAL1 and CLOCK, form the positive arm of transcriptional/translational feedback loops by heterodimerizing and binding to the E-box element on circadian target genes to activate transcription, including period (PER) (homologs: 1-3), cryptochrome (CRY) (homologs: 1 and 2), retinoid-related orphan receptor (ROR), Rev-Erb, and other genes in output pathways. The negative feedback loop is created by the complex formed by PER and CRY, which blocks BMAL1/CLOCK-driven transcription. The expression of the BMAL1 gene is modulated by ROR and Rev-Erb, respectively, which also affects the activity of the loops. Other circadian-controlled genes, such as AANAT, the primary enzyme for melatonin production, are also rhythmically expressed in response to the circadian clock genes. Controlled circadian cycles consequently influence the rhythmicity and expression level of melatonin, which also influences the quantity and quality of sleep. Dysregulation of melatonin will cause sleep problems and aberrant miRNA levels as well as gene expression patterns. Genes linked to ASD may have altered expression levels due to abnormal miRNA expression. Alterations in brain metabolism may also be brought on by ASD-mediated altered gene expression, including changes in miRNA levels. Created with BioRender.com.

There have been several studies that have reported disrupted circadian rhythms in individuals with ASD. These disruptions can manifest in a variety of ways, such as difficulty falling asleep, difficulty staying asleep, and an overall change in the timing of sleep. Additionally, some research has found that individuals with ASD may have a reduced sensitivity to light, which can further disrupt their circadian rhythms. This is associated with the autonomic nervous system and circadian rhythms. Sleep disorders are correlated with the severity of ASD symptoms. *SHANK3*, a candidate gene for predicting ASD risk, has been shown to regulate the expression of circadian and sleep transcription factors such as PER3, Bhlhe41, Hlf, Tef, and Nr1d1 in mice, providing molecular basis for sleep problems in patients with ASD (Ingiosi et al. 2019). In addition to genetic factors, the circadian rhythm-controlled hormones, such as melatonin and cortisol, affect ASD. Melatonin disruptions, especially in patients with ASD, appears to represent changes associated with sleep disorders, gastrointestinal motor changes, behavioral and emotional defects, and sensory protein dysfunctions (Dell'Osso et al. 2022) (Figure 1).

Sleep disorders are more common in children with ASD than in those with developing and intellectual disabilities (Yenen and Çak 2020). Most studies agree that sleep problems are associated with behavioral disorders in children with ASD (Malow et al. 2006). While less than 30% of the actual child population have sleep problems, approximately 50–80% of children with ASD are estimated to have sleep problems. Recent studies have shown abnormal melatonin secretion in children with ASD, suggesting that a disrupted internal clock and a dysregulated 24-hour cycle may be associated with ASD. It is found that children with ASD had significantly lower melatonin levels with also altered melatonin synthesis compared to typically developing children, which may contribute to sleep difficulties (Melke et al. 2008; Doyen et al. 2011; Tordjman et al. 2013; Gringras et al. 2017; Wu et al. 2020b). And children with ASD had a delay in the timing of their melatonin onset, which may contribute to difficulty falling asleep (Rossignol and Frye 2011; Martinez-Cayuelas et al. 2022). Indeed, studies using different methods and independent autism samples indicate that abnormally low melatonin levels are a frequent characteristic of ASD (Ritvo et al. 1993; Tordjman et al. 2005; Melke et al. 2008; Tordjman et al. 2012). Melatonin, which affects these circadian rhythms, is the backbone of sleep regulation. It also plays an important role in neurodevelopment. During pregnancy, the establishment of regular sleep patterns and circadian rhythms of the fetus, which are crucial for appropriate neurodevelopment, is dependent on the melatonin hormone (Voiculescu et al. 2014). The melatonin crosses the placenta into the fetal circulation and transmits photoperiodic information to the fetus. A fetus’s neurodevelopment is disrupted by an aberrant sleep pattern, such as disturbed REM sleep brought on by an irregular melatonin level, rhythm, or sensitivity (Morrissey et al. 2004; Tamura et al. 2008; Voiculescu et al. 2014; Jin et al. 2018). Therefore, abnormal melatonin secretion is associated with neurodevelopmental abnormalities, including circadian disorders and ASD (Jin et al. 2018). And it was also found that children with ASD had lower cortisol levels in the morning and higher at night compared to typically developing children, which may indicate a disruption in the HPA axis and the circadian rhythm with sleep difficulties (Gunnar and Vazquez 2001; Corbett et al. 2008; Corbett et al. 2009; Hughes 2009).

Some genetic factors may predispose some individuals more with ASD than others. In one study, the researchers screened single nucleotide polymorphisms in eleven clock/circadian-controlled genes in 110 individuals with ASD and their parents. Significant allele associations were detected for clock genes *Per1* and *Npas2* (Nicholas et al. 2007)*.* It was found that a specific clock gene called BMAL1 mutant mice showed autistic-like behavior with disruptions in the circadian rhythm (Liu et al. 2022a). Indeed, missense mutation of BMAL1 was found in ASD and haploinsufficiency of BMAL1 caused altered circadian rhythm and autism-like behavior in mice (Yang et al. 2016; Singla et al. 2022). Additionally, clock genes period1 (PER1) and cryptochrome1 (CRY1) were disrupted in a mouse model of Fragile X Syndrome (FXS), which is one of the most known genetic causes of autism (Lewis et al. 2007; Zhang et al. 2008; Spencer et al. 2011; Dolan et al. 2013; Sare et al. 2017).

In recent studies, microRNAs (miRNAs) have attracted attention as important biomarkers for identifying ASD (Hu et al. 2017; Ortega et al. 2021). miRNAs function as gene expression regulators. They are a common class of endogenous short, non-coding RNAs that primarily regulate the expression of protein-coding genes at the mRNA level via translational repression and/or degradation of their target mRNAs (Iwakawa and Tomari 2015; O'Brien et al. 2018). The Argonaute (AGO) proteins attach to the short (20–22 nucleotide [nt]) miRNAs, which then direct the AGO-associated RNA-induced silencing complex (RISC) to specific mRNA targets (O'Brien et al. 2018; Muller et al. 2019). It was observed that >60% of protein-coding genes are under selective pressure to retain pairing with miRNAs (Shu et al. 2017). AGO2 then facilitates the miRNA-dependent interaction of the RISC complex with mRNAs. This suggests that the majority of protein-coding genes are very susceptible to regulation by miRNAs. Recent evidence suggests that the genes regulating the circadian rhythm are regulated by miRNA that consists of 20–22 short, small non-coding nucleotides (Kim et al. 2009). The functions of miRNAs are diverse and include mRNA stability, control of translation, and regulation of heterochromatin formation. Through a wide range of miRNA functional roles across the genome and transcriptome, miRNAs are involved in almost all biological processes, including during the developmental stages, cell differentiation, cell proliferation, cell death, metabolic regulation, transfusion silence, and antiviral defense. A number of miRNAs have aberrant expression patterns in ASD, and miRNAs are indeed involved in the regulation of ASD-related genes (Kinoshita et al. 2020).

Therefore, ASD and circadian rhythm are closely related, and this study aimed to investigate the association between ASD and circadian rhythm using miRNAs.

## MicroRNAs influencing both circadian rhythm and ASD

### Circadian rhythm-related miRNAs

The mammalian tissues and cells have autonomous 24-hour cycle oscillators to generate circadian rhythm. The circadian rhythm is generated by a transcription-translation feedback loop that is combined with post-transcription and post-translational modifications. The mechanism of a circadian clock system consists of several feedback loop systems that include transcription and translation steps. The first loop includes positive elements, such as CLOCK and BMAL1. The heterodimers of CLOCK and BMAL1 activate the transcription of target genes, including the E-box cis-modulating enhancer sequences, period (*PER1-3*), and cryptochrome (*CRY1, 2*) genes of the *CLOCK* family. Negative feedback is achieved by the PER and CRY heterodimer proteins acting on the CLOCK/BMAL1 complex in the nucleus to inhibit its transcription. CLOCK can be replaced by the neuron PAS protein 2 (NPAS2), an analog of CLOCK that dimerizes with BMAL1 to form transcriptionally active complexes (Landgraf et al. 2016). NPAS2 can compensate for the loss of CLOCK in peripheral cells and SCNs. The second loop includes retinoic acid-related orphan nuclear receptors (RORs), which work through enhancers of the ROR reaction element (RORE) and REV-ERB (Takahashi 2017).

These circadian clock genes are regulated at different levels including transcriptional, translational, post-transcriptional, and post-translational modifications (Treiber et al. 2019). Several miRNAs, in particular, target circadian-controlled genes and regulate their expression (Table 1).

**Table 1.** MicroRNAs that directly target circadian clock genes.

miRNA	Target Gene	Function
miR-107 (Daimiel-Ruiz et al. 2015), miR-124 (Li et al. 2013), miR-17-5p (Gao et al. 2016), miR-365 (Na et al. 2009), miR-181 (Ding et al. 2016), miR-200b (Na et al. 2009), miR-146b (Kochan et al. 2015), miR-, miR-182 (Na et al. 2009), miR-219*, miR-132*	CLOCK	­ Control of Cell 24 Hour Cycle Rhythm ­ The Growth and Branch Formation of Neural Axons ­ The melatonin synthesis and AANAT-mRNA level are controlled
miR-219 (Arnes et al. 2019) has-Let-7 (Chen et al. 2014), miR-10 (Lundy 2018; Horii et al. 2019), miR-135b (Jiang et al. 2018), miR-142 (Zhou et al. 2021), miR-155 (O'Connell et al. 2008; Woodbury et al. 2015), miR-27b-3p (Zhang et al. 2016), miR-494 (Shende et al. 2014), miR-191 (Chu and Zhao 2013), miR-202 (Elzein and Goodyer 2014), miR-376b (Oyama et al. 2017),	BMAL1	- Promotion of ALS pathogenesis - Inflammatory response regulation in the brain - Target control of ASD-induced genes
miR-181a (Zhang et al. 2019), miR-27a (Zhang et al. 2019), miR-19b (Uwatoko et al. 2019), miR-503-5p (Zhang et al. 2019), miR-183-5p (Zheng et al. 2018), miR183-5p (Kinoshita et al. 2020)	RORa	- Tumor Repression
miR-328 (Na et al. 2009), miR-34a (Uwatoko et al. 2019), miR-29b (Hong et al. 2014; Zhao et al. 2014)	PER 1	- Relevant to Cerebellar Degeneration Disorder Sleep Patterns - Dopaminergic neuron loss - Retinal Microvascularization and Neurological Defects - Induced tau hyperphosphorylation - Dopaminergic neuron loss - Cancer cell growth inhibitor
miR-34a (Uwatoko et al. 2019) miR-192 (Zhou et al. 2021), miR-194 (Zhou et al. 2021), miR-24 (Yoo et al. 2017), miR-25 (Park et al. 2020), miR-30 (Du et al. 2014), miR-92a (Bhatwadekar et al. 2015)	PER 2	
miR-146b (Na et al. 2009), miR-103 (Hong et al. 2014), miR-29b-3p (Dalgaard et al. 2022), miR-29b (Hong et al. 2014; Chen et al. 2014)	PER 3	
miR-146b (Na et al. 2009) miR-185 (Lee et al. 2013), miR-383 (Mehta and Cheng 2013)	CRY 1	- Improves dietary disorders (Schroeder et al. 2018) - TrkB-T1 regulation (Serafini et al. 2014) - Cancer cell growth inhibitor
miR-7-5p (Tang et al. 2020), miR-181d (Guo et al. 2017), miR-27a-3p (Ren et al. 2021)	CRY 2	
miR-181a-5p (Knarr et al. 2019b)	CRY 3	
miR-324-3p (Liu et al. 2022b)	REV-ERB	- Tumor suppressor
miR-208 (Na et al. 2009)	CKle	- Bio marker
miR-146b (Na et al. 2009), miR-483 (Na et al. 2009), miR-324-3p (Na et al. 2009), miR-350 (Na et al. 2009)	Timeless	- Facilitating apoptosis - Upregulation in the serum of a person with poor sleep quality
miR-208 (Na et al. 2009), miR-520 g (Na et al. 2009), miR-199b (Yuan et al. 2020)	NPAS2	- Involved in oxygen metabolism and regulation - Vascularization
miR-7 (Qiu et al. 2019), miR-325-3p (Yang et al. 2017); miR-483 (Alamdari et al. 2021), miR-328a-3p (Zheng et al. 2022)	AANAT	- Controlling Melatonin Synthesis

Notes: Clock genes-targeting human miRNAs that have specific effects on the brain and nerve cells were classified by target genes and functions. The annotated miRNA regulates the expression of the clock gene, and the function here refers to the direct or indirect outcome. In the case of the BMAL1 function, due to the interdependence of circadian clock genes, malfunction of BMAL1 will result in dysregulation of other clock genes as PER2, PER3, CRY1, and CRY2. Indeed, it has been noted that some ASD patients have mutations in these circadian clock genes (Yang et al. 2016; Charrier et al. 2017). BMAL1 also controls the expression of ASD-causing genes such mTOR (Ganesan et al. 2019; Singla et al. 2022), Reelin (RELN) (Ali et al. 2020), MeCP2 (Nagarajan et al. 2006; de Paz et al. 2015), NPAS2 (Charrier et al. 2017), TIM (Yang et al. 2016; Charrier et al. 2017), DBP (Yang et al. 2016; Charrier et al. 2017), and Ck1ϵ (Yang et al. 2016; Charrier et al. 2017). Synaptic genes including SHANK3, NLGN3, NLGN4, and NRXN1 may be affected by genes that regulate circadian rhythms like BMAL1 and genes associated to melatonin synthesis, while no direct correlation has been observed (Sarowar et al. 2016; Charrier et al. 2017). *, predicted by TargetScan and miRDB.

miRNA expression also can be controlled by the circadian system. CLOCK-BMAL1 directly regulates the expression of a number of miRNAs, including miR-219 (Cheng et al. 2007; Kojima et al. 2011). Additionally, in reaction to light, some miRNA, including miR-132, are activated by mitogen-activated protein kinase and a cAMP response element binding-dependent mechanism (Cheng et al. 2007; Kojima et al. 2011).

Direct transcriptional activation of miR-219 by CLOCK protein results in rhythmic miR-219 expression (Cheng et al. 2007). The level of miR-219 expression affects the length of the mouse circadian period (Cheng and Obrietan 2007; Cheng et al. 2007). The miR-183/96/182 cluster is highly expressed in the nervous system, especially in the sensory organs such as the inner ear and retina (Fan et al. 2017). Retinal degeneration occurs when the members of the miR-183/96/182 cluster are disrupted. The retina is not only a sensory organ but also a self-maintained 24-hour cycle clock. It is essential in setting the circadian rhythm as it is the only organ with light receptors and transports the signal to the SCN via the optic nerves (Zhou et al. 2021). Additionally, a photic signal induces miR-132 transcription in a CREB-dependent manner, which is followed by circadian time-dependent expression (Cheng et al. 2007). Translation regulatory genes and chromatin remodeling genes such as MECP2 are examples of mRNAs that miR-132 specifically targets (Alvarez-Saavedra et al. 2011). Through chromatin remodeling and protein translation, miR-132 then fine-tunes circadian rhythm entrainment. As a result, miR-132 has the potential to modify SCN’s capacity to relay photic signals for the production of other circadian-controlled genes.

In contrast, some miRNAs affect sleep by targeting the pineal glands of the brain. miR-132 is also associated with processes related to sleep control. In addition to miR-132, various miRNAs, such as miR-138, miR-let-7b, and miR-125a-5p, affect sleep in the brain (Davis et al. 2012). There are also reports that naturally occurring mutations of miR-182 target circadian-controlled genes, causing insomnia. Because genetic variations in the form of precursors of miR-182 cause major depression in patients with late insomnia, these miRNAs may be involved in 24-hour cycles and sleep functions (Saus et al. 2010). In addition, abnormal miRNAs such as miR-130a, miR-26a, miR-30c, and miR-let-7f are commonly detected in the plasma of patients with narcolepsy and idiopathic hypersomnia (Holm et al. 2014).

Melatonin is an important hormone that affects various bodily processes in the body and is secreted by the pineal gland in the brain (Kennaway and Wright 2002; Cipolla-Neto and Amaral 2018). Melatonin synthesis and secretion by pineal gland are under SCN control and are influenced by the circadian rhythm. Melatonin is synthesized from serotonin after the enzymatic actions of aryl alkyl amine N-acetyl transferase (AANAT) and acetylacetone O-methyltransferase (ASMT) (Rath et al. 2016). AANAT is the rate-limiting enzyme in melatonin synthesis pathways, and the circadian rhythm controls its expression (Coon et al. 1995). Melatonin synthesized by AANAT has a regulatory effect on certain pathways leading to diseases/disorders, such as cancer, brain conditions, liver fibrosis, ASD, and atherosclerosis, through functional interactions with specific miRNAs (Su et al. 2018). Several miRNAs, such as miR-483, can affect AANAT mRNA stability, AANAT activity level, and ultimately, melatonin level. The decrease in AANAT activity induced by miR-483 may reflect a decrease in AANAT protein expression (Coon et al. 1995).

Circadian-controlled genes inversely regulate the expression of several miRNAs (Ma et al. 2020). For example, 24-hour cycle initiators CLOCK and BMAL1 show a negative correlation with the 24-hour cycle expression patterns for miR-181d and miR-191. In contrast, the 24-hour periodic inhibitors PER, CRY, CKIe, and Rev-erb are positively correlated with miR-181d and miR-191 (Na et al. 2009).

### ASD-related miRNAs

The majority of ASD cases appear to be brought on by mutations in any of the ASD risk genes and arise early in embryonic development (Fernandez and Scherer 2017). More than 100 gene mutations associated with brain development and neuronal activity have been identified in patients with ASD and are believed to be biomarkers for ASD (Liu et al. 2014; Herrero et al. 2020). ASD-related genetic variants have been identified in miRNA, miRNA biosynthesis, and miRNA target genes (Hu et al. 2017). And Table 2 provides an overview of miRNAs exhibiting aberrant expression patterns linked to ASD (Table 2).

**Table 2.** Upward or down-regulated microRNAs in ASD patients.

Levels in Autism	UP	DOWM	Irregular
miR	let-7a-1, let-7a-2, let-7a-3, let-7f-1, let-7f-2, let-7g, let-7g-3p, let-7i, miR-101-3p, miR-106-5p, miR-106a, miR-106b, miR-106b-5, miR-10a-5p, miR-1246, miR-1248, miR-1249, miR-127-3p, miR-1273c, miR-1277-3p, miR-1277-5p, miR-129-2-3p, miR-130a, miR-130b-5p, miR-132, miR-133b, miR-136, miR-140, miR-140-3p, miR-142-3p, miR-142-5p, miR-144-3p, miR-146a, miR-146a-5p, miR-146b, miR-148a, miR-148a3p, miR-150, miR-153, miR-155-3p, miR-155-5p, miR-16-2, miR-17, miR-181b-3p, miR-181d, miR-182, miR-186, miR-188-5p, miR-189, miR-18b-3p, miR-18b-5p, miR-190, miR-191-5p, miR-193b, miR-195b-5p, miR-199b, miR-19a-3p, miR-19b-3p, miR-20b-5p, miR-21-3p, miR-21-5p, miR-210-5p, miR-211, miR-218-2-3p, miR-218-5p, miR-219, miR-219-5p, miR-221-3p, miR-222-5p, miR-223-3p, miR-223-3p, miR-223-5p, miR-2277-5p, miR-23b, miR-2467-5p, miR-26a-2, miR-27a-3p, miR-28-5p, miR-301a, miR-30b, miR-30c-1, miR-30c-2, miR-30d-3p, miR-3168, miR-32-5p, miR-326, miR-335-3p, miR-335-3p, miR-338-5p, miR-34b-3p, miR-34c-3p, miR-3529-3p, miR-3613-5p, miR-363-3p, miR-367, miR-374b, miR-374b-5p, miR-379-5p, miR-381, miR-3938, miR-424-3p, miR-4270, miR-4299, miR-432, miR-4436a, miR-4443, miR-4489, miR-449b-5p, miR-450b-5p, miR-451, miR-4516, miR-455, miR-4669, miR-4705, miR-4709-3p, miR-4721, miR-4728-5p, miR-4753-5p, miR-4788, miR-483-5p, miR-494, miR-495, miR-518a, miR-520b, miR-532-5p, miR-539, miR-550, miR-557, miR-564, miR-5739, miR-574-5p, miR-575, miR-6086, miR-6125, miR-620, miR-628-5p, miR-629-5p, miR-642a-3p, miR-642a-5p, miR-642b-5p, miR-651-5p, miR-652, miR-663, miR-664-3p, miR-664a-3p, miR-665, miR-6723-5p, miR-7-5p, miR-7-5p, miR-708-5p, miR-766-3p, miR-8052, miR-874-3p, miR-921, miR-130a-3p, miR-132-5p, miR-138-1-3p, miR-139, miR-199a-5p, miR-23a-3p, miR-34c-5p, miR-3607, miR-3620-3p, miR-365a-3p, miR-425-3p, miR-887-3p, miR-92a-2-5p, miR-98	let-7a-5p, let-7b, let-7b-5p, let-7c-5p, let-7d-5p, let-7f-5p, let-7i-3p, miR-301a-3p, miR-1, miR-101-1,miR-103, miR-103a-1, miR-103a-2, miR-107, miR-1228-3p, miR-125b-2-3p, miR-125b-5p, miR-126, miR-128, miR-129, miR-142, miR-145, miR-146a, miR-148a-5p, miR-151a, miR-151a-3p, miR-15a, miR-15a-5p, miR-15b, miR-16-1, miR-16-5p, miR-181a-1, miR-181a-2, miR-181b-5p, miR-183-5p, miR-185, miR-186, miR-186-5p,miR-18a, miR-191, miR-193b-3p, miR-194, miR-195, miR-195-5p, miR-197-5p, miR-19a, miR-19b-1, miR-204-3p, miR-205, miR-20a, miR-20a-5p, miR-20b-3p, miR-21, miR-211-5p, miR-212, miR-214-3p, miR-221, miR-222, miR-23a, miR-23a-3p, miR-23b, miR-25, miR-27a, miR-27a-3p, miR-27b, miR-28-3p, miR-297, miR-29a, miR-29b, miR-29b-1, miR-29b-2, miR-29c-5p, miR-3064-5p, miR-30e, miR-30e-5p, miR-3135a, miR-32-5p, miR-320,miR-328, miR-328-3p, miR-342,miR-346, miR-34a-5p, miR-34c-5p, miR-3613-3p, miR-363, miR-3674, miR-3687, miR-376a-AS, miR-3909, miR-3935, miR-3960, miR-409, miR-423, miR-423-5p, miR-431, miR-433, miR-434, miR-4433b, miR-4436b-5p, miR-451, miR-4665-5p, miR-4700-3p, miR-4742-3p, miR-487b-3p, miR-489, miR-491-5p, miR-500a-5p, miR-504-5p, miR-5096, miR-519c, miR-524, miR-5701-1, miR-5701-2, miR-572, miR-574-3p, miR-576-5p, miR-587-3p, miR-589-3p, miR-598, miR-654-5p, miR-656, miR-663a, miR-664b-3p, miR-671-3p, miR-6799-3p, miR-6849-3p, miR-7, miR-7706, miR-92, miR-92a-3p,miR-92b-3p, miR-93, miR-95,miR-96-5p, miR-99a-5p, miR-101-2, miR-103a-3p, miR-16-2,miR-19b-1-5p, miR-193a-5p, miR-199a-5p, miR-199b,miR-19b-2, miR-19b-3p, miR-27a-3p, miR-29c, miR-30e, miR-376c, miR-625	miR-103a-3p, miR-107, miR-119b-5p, miR-132-3p, miR-145-5p, miR-148b, miR-199a-5p, miR-230a, miR-320a, miR-3609, miR-423-5p, miR-424-5p, miR-451a, miR-484, miR-486-3p, miR-619-5p, miR-628-5p, miR-93-5p, miR-940
References	Mor et al. (2015); Wu et al. (2016); Nguyen et al. (2018); Abu-Elneel et al. (2008); Ander et al. (2015); Yu et al. (2018); Talebizadeh et al. (2008); Sarachana et al. (2010); Ghahramani Seno et al. (2011); Mundalil Vasu et al. (2014); Huang et al. (2015); Hicks et al. (2016); Jyonouchi et al. (2017); Toma et al. (2015); Williams et al. (2019); Nguyen et al. (2016); Tonacci et al. (2019); Mundalil Vasu et al. (2014); O'Brien et al. (2018)	Mor et al. (2015); Wu et al. (2016); Abu-Elneel et al. (2008); Ander et al. (2015); Yu et al. (2018); Talebizadeh et al. (2008); Sarachana et al. (2010); Ghahramani Seno et al. (2011); Mundalil Vasu et al. (2014); Huang et al. (2015); Hicks et al. (2016); Jyonouchi et al. (2017); You et al. (2019); Mundalil Vasu et al. (2014); O'Brien et al. (2018)	Halepoto et al. (2014); Sabaie et al. (2021)

Note: Human miRNAs were classified according to each level in patients with ASD.

Many studies have argued for the genetic pathologies of ASD, particularly those associated with synaptic cell adhesion molecules NLGN3, NLGN4, and NRXN1, and the postsynaptic scaffold protein SHANK3. One of the upstream factors that can control these genes might be circadian rhythm-controlled gene (Sarowar et al. 2016).

miR-146 is a strong candidate as an ASD biomarker since it has altered expression across a variety of tissues in individuals with autism. miR-153 is an important miRNA extensively studied in ASD, and it is shown that LEPR is a target gene of miR-153 in autism. miR-34 is another extensively studied miRNA in ASD. Recent studies have reported its vital role in neuronal development and disorders. Moreover, it has a strong influence on the regulation of *MET*, which has been reported as a risk gene in ASD. Other miRNAs, such as miR-106, miR-130, miR-320, and miR-451, have also shown altered expression levels in the brain and biofluids of individuals with ASD. The target genes (e.g. *TGF-β, MECP2, NLGN3, PTEN, AUTS2, TSC1, SLITRK, NFkB, MAPK, AKT, ERK*, and *VEGF)* of these miRNAs have been implicated in the pathogenesis of ASD as well as in neurodevelopment and neuronal functions (Vasu et al. 2019). hsa-miR-106b has been shown to be associated with autism and a variety of brain disorders (Zadehbagheri et al. 2019). Among the aforementioned ASD risk genes, *TNRC6B, PTEN, AGO1, AGO2, SKI*, and *SMAD4* were the most commonly expressed, and are targeted by miR-92a-3p miRNAs for their regulation. In addition, this miRNA is involved with ASD risk genes and in a variety of pathways, including circadian rhythms, long-term depression, mTORs, and estrogen signaling pathways. miR-7-5p inhibits the expression of the ASD-related gene *PAX6*, an important transcription factor in neuronal tissue development that regulates dopaminergic neuronal differentiation (de Chevigny et al. 2012). Overexpression or inhibition of miR-7 and miR-504 also modulates the expression of the ASD risk gene *Shank3* and affects the development of hippocampal neurons (Choi et al. 2015). miR-155 adversely affects the brain–blood-barrier function during neuroinflammation by targeting cell–cell complex molecules, such as AA2, claudin-1, and molecules that are critical in cell-to-extracellular matrix (ECM) interactions, including dedicators of cytokinesis 1 and syntenin-1 (Lopez-Ramirez et al. 2014). This means that miRNAs may contribute to the dysfunction of adherent junctions, the brain–blood barrier, and intestinal epithelial barrier in ASD (Fiorentino et al. 2016). Cell nutrient and energy detection by mTOR signaling regulate almost every aspect of metabolism and mitochondrial biosynthesis and play an important role in glucose homeostasis, lipid homeostasis, immune function, brain function, and cancer (Saxton and Sabatini 2017). miR-107 and miR-103 regulate insulin signaling and glucose homeostasis to help detect cellular nutrients and energy via mTOR signaling. The mTOR signal contains several ASD risk genes, including *IGF1, MTOR, PIK3R2, PTEN, RHEB, TSC1*, and *TSC2.* This may partly explain the causes of mitochondrial dysfunction and various clinical symptoms in ASD (Trajkovski et al. 2011).

Among the miRNAs mentioned above, those listed in Table 3 are involved in both circadian rhythms and ASD. To explain the selected miRNAs, the most downregulated miR-219 in ASD patients was found to be involved in the control of the circadian rhythm in the SCN. miR-219 can directly target polo-like kinase 2 (PLK2), and PLK2 overexpression can reduce synaptic strength and neuroexcitability, leading to synaptic dysfunction in patients with ASD (Sarachana et al. 2010; Li et al. 2022). miR-219 is a target of the master circadian regulators CLOCK and BMAL1 (brain and muscle ANT-like 1) complexes, shows strong circadian rhythmic expression, and finely adjusts the length of the circadian rhythm in mice (Cheng et al. 2007; Kojima et al. 2011). In the case of miR-29b, it can influence the expression of Per1 and Per3 by directly targeting their 3′UTR (Mellios and Sur 2012; Hong et al. 2014; Zhao et al. 2014; Wu et al. 2020).

**Table 3.** MicroRNAs that are involved in the circadian rhythm and are abnormally controlled in patients with ASD.

miRNA	Target clock gene	Levels in ASD	Function	References
miR-107	CLOCK	Up	Alzheimer’s induction factor	Daimiel-Ruiz et al. (2015); Nelson and Wang (2010)
miR-199b		Up	Tumor, obstructive apnea induction	Yuan et al. (2020)
			NPAS2 overexpression reduces oxidative phosphorylation	
miR-17-5p		Irregular	Control the expression of a clock	Gao et al. (2016)
miR-211		Down	Contributes to the cell survival of Burkitt’s lymphoma	Bu et al. (2018)
miR-219*		Up	Steroid hormone metabolism and receptor signaling gene regulation	Sarachana et al. (2010)
miR-132*		Down	Important for neurodifferentiation, maturation, and functional regulation and is extensively involved in axonal growth, nerve migration, and plasticity	Cheng et al. (2007); Duffield et al. (2009); Sarachana et al. (2010)
miR-10	BMAL 1	Up	Downregulation of Bmal1 expression	Lundy (2018); Horii et al. (2019)
			Abnormal liver metabolism, cirrhosis induction	
miR-142-3p		Up	Regulators of SIRT1	Matamala et al. (2018); Raheja et al. (2018)
miR-155		Up	Effect of Immunomodulation and Neuroinflammatory Induction in Brain Tissues in ASD Patients	Woodbury et al. (2015); Testa et al. (2017)
has-Let-7		Irregular	The various TGF-beta signaling like SMAD4 is controlled	Chen et al. (2014); Baranova et al. (2021)
miR-494		Up	Anti-apoptotic protein survivin is targeted to weaken vesicle stress response	Shende et al. (2014); Chatterjee et al. (2020)
miR-19b	RORa	Irregular	A major regulator of circadian transcripts CLOCK and ROR	Uwatoko et al. (2019); Fernández-Santiago et al. (2015)
miR-27a		Down	Control of insulin-like growth factor-1	Wrigley et al. (2017)
miR-29b	PER 1/2/3	Up	Related to the modulation of synapses and circadian rhythm signals	Dumortier et al. (2013); Liang et al. (2013); Seeburg and Sheng (2008)
miR-181d		Up	Control of the Per3 of epileptic Cells and Cry1 in the Renal Cycle Rhythm	Knarr et al. (2019b); Knarr et al. (2019a)
miR-103		Down	An important role in brain development and function	Hong et al. (2014); Mauvoisin et al. (2014)
miR-30a		Up	Tumor suppressor	Chen et al. (2013); Martinez et al. (2011)
			Cell cycle progression control	
miR-142-3p	Reb-erb	Up	Gene regulation associated with neurological function and disorder	Shende et al. (2014); Sarachana et al. (2010)

Notes: Human miRNAs that appear to affect both circadian rhythms and ASD, are summarized according to circadian rhythm target genes and the level and function of ASD. *, predicted by TargetScan and miRDB.

## Discussion

In this review, we have attempted to explain the effect of circadian rhythm on the expression of ASD-related genes through miRNAs. Circadian rhythms affect various processes such as growth, immunity, sleep patterns, and ASD. When the circadian-controlled gene that controls the circadian rhythm is mutated, the circadian rhythm can be disrupted, causing sleep disturbances. Many patients with ASD suffer from sleep disorders, and there have been studies that show that there is limited production and secretion of melatonin in their brains (Melke et al. 2008; Doyen et al. 2011; Rossignol and Frye 2011; Gringras et al. 2017; Wu et al. 2020; Martinez-Cayuelas et al. 2022). This is an example of how ASD is linked to abnormal sleep patterns and melatonin expression, and it could be an example of the relationship between circadian rhythm and ASD.

We hypothesized that ASD might be closely associated with a circadian rhythm disorder via miRNAs. In this work, we found that a number of miRNAs, including miR-219, miR-132, and miR-146, may serve as crucial linkages for circadian rhythm disruption in ASD patients. The miR-146 family consists of the two evolutionarily conserved miRNAs miR-146a and miR-146b (Williams et al. 2008; Matsumoto et al. 2016). Since they are expressed in neurons, miR-146a and miR-146b have a role in neuronal development and the control of inflammation in the nervous system (Nguyen et al. 2018; Chithanathan et al. 2022). According to one study, gene networks that miR-146a targets are connected to ASD and can be utilized to predict the type and severity of ASD expression in addition to just diagnosing the ASD presence (Nguyen et al. 2018). The circadian gene is also expected to have an impact on the expression of miR-146a (Wang et al. 2014). Other research has suggested that the circadian genes PER3, CRY1, and Timeless may be targeted and regulated by miR-146b (Dell'Osso et al. 2022). In conclusion, miR-146a and miR-146b expression can be influenced by circadian rhythms and is connected to ASD and neural development.

As summarized in Table 3, some miRNAs that control specific genes of the circadian clock system are also expressed abnormally in patients with ASD at the same time. However, we also believe that miRNAs that are transcriptionally regulated by the circadian rhythm or not mentioned in Table 3 can directly or indirectly affect the expression of ASD inducers. Based on this, focusing on aspects of the circadian clock system, recent studies have shown that RORA-deficient mice exhibit limited behavior similar to mice with ASD, such as limited maze patrols, abnormal spatial learning, reduced search, and patience, compared to wild-type mice (Goodall and Gheusi 1987; Nguyen et al. 2010). RORA genes are dynamically regulated by several miRNAs, and miRNA-mediated RORA gene regulation may also affect ASD. For example, miR-18a negatively regulates RORA expression by binding to RORA’s 3′-UTR (Jiang et al. 2020), and it could also be an inducer of ASD. In conclusion, miRNA, which is transcriptionally regulated by the regulation of the clock gene of the circadian rhythm, might have a considerable effect on ASD. In addition to the known circadian-controlled or clock-gene-regulating miRNAs, additional miRNAs could be found, and their target genes and functions could be identified. The new targets and functions of previously reported miRNAs should also be investigated.

Although several miRNAs have been proposed to explain the association between circadian rhythm and ASD, studies have shown that ASD can be caused by various miRNAs. And in respect with ASD-related genes, behavioral tests using the SHANK2-KO mouse model with exon 6–7 deleted showed a decrease in interaction and social communication, memory deficit and spatial learning, hyperactivity, and anxiety-related behavior. This provides evidence that SHANK2 can cause ASD (Schmeisser et al. 2012). In this SHANK 2 gene, a single miR-137 binding site was identified: upregulation of miR-137 decreased SHANK2 expression level. miR-137 overexpression can induce ASD by downregulating SHANK2 (de Sena Cortabitarte et al. 2018). Furthermore, miR-137 has a potential association with circadian rhythm as well as with ASD. There have been studies reporting that miR-137 regulates Hypocretin (Hcrt) expression in the Hcrt neuronal cells that inhibits awakening. Hcrt neuropeptides regulate sleep and awakening stability, and Hcrt’s disorder can cause sleep disorders. Conversely, downregulation of miR-137 increases arousal in mice. The interaction between miR-137 and Hcrt is preserved across mice and humans, and studies have shown that miR-137 is genetically related to human sleep time (Holm et al. 2022). Another study found that miR-137 is involved in neuroplasticity by partially regulating glucocorticoid receptor-dependent signaling (Davis et al. 2012). Glucocorticoids form part of the awakening hormone cortisol that can affect the control of the circadian rhythm (Chung et al. 2011). In conclusion, miR-137 is a potential candidate that has the potential to affect gene expressions related to ASD, thereby, leading to ASD and to control sleep-waking rhythms to affect the circadian rhythm. Finding novel candidates such as miR-137 likely to be associated with circadian rhythm and ASD will be a challenge ahead.

miRNA transcription, Drosha and Dicer action, and RISC loading are important processes in miRNA production, and various factors promote, assist, or inhibit these processes (Treiber et al. 2019). In addition to studies that identify the function of miRNAs, recent studies have focused on regulators of miRNA expression (Debnath et al. 2017). Among various studies on miRNA-modulating substances, phytochemicals have been reported to play an important role in regulation of miRNA expression associated with changes in carcinogens, tumor inhibitors, and cancer-related protein expression. Therefore, identifying phytochemicals that can control the expression of miRNAs targeting the circadian clock and ASD-related genes might be valuable for future research. Further studies are needed to investigate the genetic effects of miRNAs associated with circadian rhythms in ASD. This can help researchers to develop treatment for ASD.
